# Astrocyte-Derived TNF-α-Activated Platelets Promote Cerebral Ischemia/Reperfusion Injury by Regulating the RIP1/RIP3/AKT Signaling Pathway

**DOI:** 10.1007/s12035-022-02942-z

**Published:** 2022-07-04

**Authors:** Wei Li, Dengping Liu, Jiaqi Xu, Jun Zha, Chen Wang, Jianzhong An, Zhanli Xie, Shigang Qiao

**Affiliations:** 1 Cyrus Tang Hematology Center, Soochow University, Suzhou, China; 2 Institute of Clinical Medicine Research, Suzhou Science & Technology Town Hospital, Gusu School, Nanjing Medical University, Suzhou, China; 3 Nursing Department, Suzhou Science & Technology Town Hospital, Gusu School, Nanjing Medical University, Suzhou, China; 4 Faculty of Anesthesiology, Suzhou Science & Technology Town Hospital, Gusu School, Nanjing Medical University, Suzhou, China

**Keywords:** Ischemic stroke, TNF-α, Platelet, Astrocytes, AP-1, RIP1/RIP3/AKT pathway

## Abstract

Ischemic stroke is a clinical syndrome caused by the disruption of blood flow into cerebral tissues and is associated with high disability and mortality rates. Studies have established the pathological role of platelets in cerebral ischemia/reperfusion (I/R) injury, although the underlying mechanism of action remains largely unclear. In this study, we created an I/R mouse model via middle cerebral artery occlusion and reperfusion (MCAO/R) and analyzed the transcriptomic profiles of the ipsilateral and contralateral cortices using RNA-seq. We found that cerebral I/R injury induced platelet invasion and accumulation in the cerebral cortex by stimulating TNF-α secretion from activated astrocytes in the ischemic region, while TNF-α expression enhanced platelet reactivity through the RIP1/RIP3/AKT pathway. Furthermore, the inoculation of TNF-α-stimulated platelets aggravated I/R injury in mice, whereas the administration of anti-TNF-α antibodies at the onset of reperfusion alleviated ischemic damage. The RNA-seq results further showed that AP-1 transcriptionally activated TNF-α in the I/R-injured cortex by directly binding to the promoter region. These findings provide novel insights into the pathological role of platelets activated by reactive astrocyte-derived TNF-α in cerebral I/R injury.

**Supplementary Information:**

The online version contains supplementary material available at 10.1007/s12035-022-02942-z.

## Introduction

Stroke is a leading cause of disability and death worldwide, and a majority of strokes are caused by cerebral ischemia, while only ∼20% of cases are the result of primary intracerebral hemorrhage [1]. Ischemic stroke is frequently associated with atherosclerosis, cardiogenic embolism, and small vessel occlusion. Most drugs that are currently used to manage ischemic stroke exert minimal curative impact while producing multiple side effects. Tissue plasminogen activator (tPA), a thrombolytic drug that breaks down the clot, is the only drug approved by the FDA for the treatment of ischemic stroke [2]. However, the therapeutic window of tPA lies within 4.5 h of the onset of stroke symptoms [3]. The administration of the drug outside of this window can result in hemorrhagic transformation and aggravate tissue damage. Therefore, the mechanisms underlying the development of ischemic stroke must be further elucidated to develop more effective therapies.

The pathophysiology of ischemic stroke includes both inflammatory and thrombogenic components [4], and studies have shown that excessive platelet aggregation is a hallmark of ischemic stroke [5]. Platelets are crucial for hemostasis and thrombosis and thus modulate innate immunity and tissue homeostasis [6]. However, blocking the final common pathway of platelet aggregation using anti-GPIIb/IIIa antibodies does not improve the outcome of acute ischemic stroke and instead elevates the risk of cerebral ischemia and mortality in a dose-dependent manner [7, 8]. Thus, cerebral ischemia cannot be explained by secondary thrombotic events in microcirculation alone.

Ischemic stroke causes an immediate local immuno-inflammatory reaction that is characterized by the activation of microglia, astrocytes and vascular endothelial cells. The infiltrating inflammatory cells and endothelial cells secrete high amounts of adhesion molecules and cytokines [9], and this inflammatory response is both a risk factor and consequence of cerebral infarction [10]. Furthermore, ischemic stroke triggers reactive astrocyte aggregation or astrogliosis in the injured marginal region, which is characterized by increased GFAP expression and hypertrophy. Astrocytes, the main inflammatory cells in the cerebral cortex, release proinflammatory factors, such as TNF-α, IL-1β and IL-6 under ischemic conditions, which promote brain injury. While astrocytes have been implicated in thrombo-inflammation, the role of platelets in this process remains undefined.

TNF-α is a proinflammatory cytokine that is primarily secreted by macrophages and monocytes. In addition, platelets can contribute to an acute ischemic episode in the brain by forming homotypic (platelet–platelet) and heterotypic (platelet–leukocyte) aggregates that cause vascular occlusion and by secreting inflammatory mediators and vasoconstrictors, such as TNF-α [4]. Dai et al. found that platelet activation depends on the RIP1/RIP3/AKT signaling pathway [11] and that TNF-α activates RIP3 by binding to its receptor (TNFR) [12]. However, it has not been clearly established whether TNF-α participates in the pathogenesis of ischemic stroke by regulating platelet reactivity.

In this study, we analyzed the pathological effect of TNF-α in a mouse model of ischemic stroke. Our findings suggest a potential novel mechanism by which activated platelets induce reactive astrocyte-derived TNF-α expression to promote cerebral I/R injury.

## Materials and Methods

### Ischemic Stroke Induction Using MCAO and Reperfusion

Male C57BL/6 J mice weighing 28–32 g at 8 weeks old were purchased from the Center for Laboratory Animals, Soochow University (Suzhou, China), and were housed at a specific pathogen-free facility under ambient conditions. All procedures were performed according to guidelines provided by the Institutional Animal Care and Use Committee of Soochow University. Ischemic stroke was induced by middle cerebral artery occlusion (MCAO) with subsequent reperfusion (MCAO/R), as previously described [13]. In brief, the mice were anesthetized with an intraperitoneal injection (i/p) of 7% chloral hydrate (5 ul/g). A 7–0 nylon suture with its tip coated with poly-lysine was inserted into the right common carotid artery and advanced through the internal carotid artery until it occluded the origin of the MCAO. Cerebral blood flow was reinstated by withdrawing the suture after 90 min of occlusion. Body temperature was maintained within the normothermic range (37 ± 0.5 °C) during and after the surgery by placing the mice on a heating pad (Institute of Biomedical Engineering, CAMS, BME-412A ANIMAL REGULATOR, 308,005,669) until the anesthetic effect wore off. Then, the mice were intraperitoneally injected with an anti-TNF-α antibody (20 μg/g, #11,969, CST, USA) or vehicle at the onset of reperfusion, and behavioral outcomes were evaluated 24 h after I/R injury. Thereafter, the mice were euthanized with an intraperitoneal injection of pentobarbital sodium, and their brains were extracted. The forebrain region was divided into five coronal sections using a mice brain matrix (Harvard apparatus), and the sections were stained with 2% 2,3,5-triphenyltetrazolium chloride (TTC, Sigma) [14] to calculate the infarction volume (% of the ipsilateral volume) using the ImageJ system (Supplemental Fig. 1).

### Behavioral Tests

Neurological function was evaluated using behavioral tests, as previously described [14]. The animals were scored for neurological deficits [15] on a 6-point scale as follows: 0, no detectable neurological deficits; 1, ptosis of the eyelid ipsilateral to the occluded MCA side and/or failure to extend ipsilateral forepaw; 2, persistently walking in large circles toward the ipsilateral side; 3, persistently walking in small circles and/or rolling over repeatedly toward the ipsilateral side; 4, lying nearly motionless on the contralateral side; and 5, death post-recovery. The cylinder test was performed to assess the asymmetric use of the forelimbs for postural support. In brief, the mice were placed inside a plexiglass cylinder (HOOFAN, Wenling, Zhejiang), and forelimb use was observed during vertical movements along the wall. The final score was calculated as (non-impaired forelimb movement – impaired forelimb movement)/(non-impaired forelimb movement + impaired forelimb movement + movement of both forelimbs) [16] with twenty movements being recorded during the 10-min test for each mouse.

### Whole Transcriptome Library Preparation and RNA-Sequencing

Total RNA was isolated from the ipsilateral and contralateral cortices (*n* = 3) using an RNeasy Plus Mini Kit (QIAGEN), and quality was assessed using an Agilent 2100 BioAnalyzer, according to the manufacturer’s instructions. Two micrograms of RNA per sample was used for library construction. Ribosomal RNA (rRNA) was extracted using an Epicenter Ribo-zero rRNA Removal Kit (Epicenter, USA), according to the manufacturer’s instructions. Subsequently, strand-specific libraries were generated based on the purified RNA using the dUTP method along with the NEB Next Ultra Directional RNA Library Prep Kit for Illumina (NEB, USA), as recommended by the manufacturer. RNA-seq was performed on the Illumina HiSeq 2000 platform, and 100-bp paired-end reads were generated based on Illumina protocol. The adapter sequences were removed from the raw data, and the individual libraries were converted into FASTQ format. Sequence reads were aligned to the mouse genome (mm10) using TopHat2 (v2.0.9), and the alignment files were reconstructed using Cufflinks (v2.1.1) and Scripture (beta2). All sequences were annotated using the RefSeq database (build 37.3). The read counts of each transcript were normalized to its length and to the total number of mapped fragments per sample and were expressed as fragments per kilobase million (FPKM). The differentially expressed genes (DEGs) between the treatment and control groups were screened for using an adjusted *P* value of < 0.05 (Student’s *t* test with Benjamini–Hochberg false discovery rate (FDR) adjustment) as the cutoff. The DEGs were functionally annotated by conducting a gene ontology (GO) analysis using the GO-seq R package [17] after correcting for gene length bias. The pathways that were significantly associated with the DEGs were identified using the Kyoto Encyclopedia of Genes and Genomes (KEGG) analysis, and the analysis was conducted using KOBAS software.

### Quantitative Real-Time PCR (qPCR)

Total RNA was extracted from the ipsilateral and contralateral cortex of three mice using a QIAGEN miRNeasy Mini kit (217,004, Qiagen, Germany) and reverse transcribed using the Takara PrimeScript™ RT Master Mix (RR036A, Takara, Japan). The cDNA templates were amplified by conducting qPCR using brilliant SYBR green PCR master mixture (4,913,914, Roche, Switzerland) in a LightCycler 480 (Roche, Switzerland). The target mRNA expression levels were normalized to that of glyceraldehyde-3-phosphate dehydrogenase (GAPDH), and the relative fold change was calculated using the 2^−ΔΔCT^ method. The primer sequences used are listed in Supplementary Table 1.

### Patients and Samples

A total of 27 ischemic stroke patients (aged 40 to 79 years), who received thrombolytic therapy alone, were recruited in this trial. These patients were confirmed by CT or MRI of the brain within 48 h of symptom onset with an elevated systolic blood pressure (BP) between 140 and < 220 mmHg. Patients with a BP ≥ 220/120 mmHg, severe heart failure, acute myocardial infarction or unstable angina, atrial fibrillation, aortic dissection, cerebrovascular stenosis, or resistant hypertension in a deep coma were excluded. In this study, 15 participants were further excluded because they did not offer blood samples, or some collected samples were hemolyzed in collections of blood samples, or we failed to determine plasma TNF-α concentrations. Fasting blood samples were collected between 2 and 7 days after thrombolytic therapy. Control plasma was obtained from heparinized blood from a healthy donor pool (five male and three female volunteers, aged 40 to 79 years). All plasma samples were frozen at − 80 °C until laboratory testing. All participants provided informed consent. This study was approved by the Ethics Committee of Suzhou Science and Technology Town Hospital.

### Primary Cortical Astrocyte Culture

Cortical astrocytes were cultured as previously described [18]. Cerebral cortices were isolated from 1- or 2-day-old C57BL/6 J mice, mechanically dissociated and digested using trypsin/DNase, and passed through a sterile 40 lm Nitex mesh. The astrocytes were suspended in DMEM/F12 (1:1) (GIBCO, 11,330) supplemented with 10% heat-inactivated fetal bovine serum (FBS; GIBCO, 10,099) and 1% 100 U/ml penicillin/streptomycin (Beyotime, C0222) and were seeded into culture dishes or plates coated with poly-L-lysine. Then, the cells were cultured at 37 °C in a humidified atmosphere with 5% CO_2_.

### Oxygen and Glucose Deprivation and Reoxygenation (OGD/R)

OGD/R was used to mimic I/R injury in vitro [18]. In brief, the cells were washed twice with PBS, and cultured in glucose-free DMEM (GIBCO, 11,966) under hypoxic conditions (95% N_2_ and 5% CO_2_) for 6 h and then in complete DMEM/F12 under normoxic conditions for 24 h. Anti-TNF-α antibodies (10 ng/ml, #11,969, CST, USA) or the vehicle was administrated during reoxygenation. The isolated supernatant was incubated with mouse platelets. Platelet aggregation, clot retraction, and spreading were measured (Supplemental Fig. 2).

### Enzyme-Linked Immunosorbent Assay (ELISA)

TNF-α levels in the culture medium, serum, and brain homogenates were measured using mouse (PT512, Beyotime, China) and human (PT518, Beyotime, China) TNF-α ELISA kits, according to the manufacturer’s instructions. The optical densities of the plates were read at 450 nm using a spectrophotometer (Bio-Rad Laboratories, Hercules, CA, USA). All experiments were performed at room temperature, and samples were analyzed in triplicate.

### Immunohistochemistry (IHC)

IHC was performed as previously described [18]. In brief, the mice were anesthetized using 4% chloral hydrate (350 mg/kg) and perfused with PBS, and then 4% paraformaldehyde was injected through the left cardiac ventricle. The brains were extracted and fixed in 4% paraformaldehyde at 4 °C for 24 h, immersed in phosphate buffer containing 20% or 30% sucrose, and sliced into 10-mm-thick sections using a cryostat. The sections were rinsed with PBS for 15 min, blocked with 1% bovine serum albumin (BSA) in 0.3% Triton X-100 for 1 h, and then incubated overnight with mouse anti-CD42d (1:100, AF6990, R&D, USA), mouse anti-TNF-α (1:100, ab1793, Abcam, USA), and rabbit anti-GFAP (1:500, AB5804, Millipore, USA) antibodies. After being washed again with PBS, the sections were incubated with Alexa Fluor 488 donkey anti-sheep IgG(H + L) (1:200, A-11015, Invitrogen, USA), Alexa Fluor 488 sheep anti-mouse IgG (H + L) (1:500, AB150113, Abcam, USA), and Alexa Fluor 647 donkey anti-rabbit IgG(H + L) (1:500, AB150063, Abcam, USA) secondary antibodies for 1 h at room temperature in the dark. Then, the sections were counterstained with DAPI (1: 10,000, D9564, Sigma), dried, and mounted on coverslips. Images were obtained under a confocal microscope (LSM 710, Carl Zeiss Co. Ltd., Oberkochen, Germany).

### Platelet Isolation and Aggregation

Mouse blood was diluted 1:5 with an anti-coagulant (65 mM Na_3_ citrate, 70 mM citric acid, and 100 mM dextrose; pH 4.4) and centrifuged at 900 rpm for 20 min to obtain platelet-rich plasma (PRP) [19]. The PRP was then loaded into a Sepharose^TM^2B column, and the platelets were eluted using Tyrode’s buffer [16]. The gel-filtered platelets were pooled, and the count was adjusted to 2.5 × 10^8^/mL in Tyrode’s buffer. Platelet aggregation was performed in a ChronoLog aggregometer (Havertown, PA, USA) using prepared washed platelets. The platelets were suspended in Tyrode’s buffer and calcium (1 mM) was added, as described previously. The percentage of aggregation was calculated after adding thrombin (1,060,240,001, Sigma, USA) or U46619 (538,994, Calbiochem, USA) using the amplitude of the tracings at 5 min, and the results were normalized to the results of the untreated control of each individual experiment.

### Platelet Spreading on Immobilized Fibrinogen

Chamber slides with microtiter wells were coated overnight with 10 μg/mL human fibrinogen (F4884, Calbiochem, USA) in 0.1 M NaHCO_3_ (pH 8.3) at 4 °C. Washed platelets (2 × 10^7^/mL) were allowed to adhere to and spread on the inner surface of the fibrinogen-coated wells at 37 °C for 2 h in the presence of thrombin (1,060,240,001, Sigma, USA). The cells were washed once, fixed, permeabilized, and stained with Alexa Fluor 488-conjugated phalloidin (A12379, Life Technologies, USA). The adherent platelets were viewed under an Olympus FluoView FV1000 confocal microscope, and images were acquired. The area traversed by each individual platelet was measured in pixels using ImageJ2x software. Ten randomly selected fields from at least three different tests were used for the statistical analysis.

### Clot Retraction

Washed mouse platelets (4 × 10^8^/mL) were resuspended in modified Tyrode’s buffer in unused aggregometer tubes and mixed with 150 μg/mL purified human fibrinogen. Clotting was initiated in the presence of 1 U/mL thrombin at 37 °C for 60 min. Clot retraction was monitored every 5 min and photographed, while clot size was measured using ImageJ2x software.

### Immunoblotting

Protein was extracted from the platelets or brain homogenates using RIPA buffer supplemented with a protease inhibitor and quantified using a bicinchoninic acid (BCA) (23,227, Thermo, USA) kit. Equal amounts of protein from each sample were separated using 8% SDS-PAGE, transferred onto a nitrocellulose membrane, and incubated overnight with antibodies targeting p-RIP1 (Ser166, 1:1000, 53,286, CST, USA), RIP1 (1:1000, ab202985, Abcam, USA), p-RIP3 (Ser232, 1:1000, ab195117, Abcam, USA), RIP3 (1:1000, ab62344, Abcam, USA), p-AKT (Ser 473, 1:1000, 4060 T, CST, USA), AKT (1:1000, 4691S, CST, USA), Fosb ( 1:1000, ab11959, Abcam, USA), Jun (1:1000, ab40766, Abcam, USA), Jund (1:1000, ab181615, Abcam, USA), Fos (1:1000, ab222699, Abcam, USA), Junb (1:1000, 128,878, Abcam, USA), and β-actin (1:5000, A5441, Sigma) in Tris buffered saline containing 0.2% Tween-20 (TBST) and 5% nonfat dry milk at 4 °C. After washing with TBST, the membranes were incubated with 1 μg/ml goat anti-rabbit IRDye 800CW or goat anti-mouse IRDye 800CW (Licor Odyssey, USA). The positive bands were detected using the Odyssey infrared imaging system (LI-COR Biosciences, USA), and signal intensity was quantified using ImageJ software and normalized to that of β-actin.

### Luciferase Reporter Assay

HEK293T cells (Cell Bank of Chinese Academy of Sciences, Shanghai, China) were cultured in DMEM supplemented with 10% FBS (HyClone, Utah, USA) at 37 °C in a humidified incubator under 5% CO_2_ (Thermo Forma Electron Co., Ohio, USA). The − 2000/ − 1 bp region of the *TNF-α* gene was cloned into the Xho I and Kpn I sites of the luciferase reporter vector (Luc), GV238. HEK 293 T cells were seeded into 96-well plates and were co-transfected with 0.1 μg TNF-α pro-Luc or empty-Luc plasmid (empty-Luc) and 0.1 μg Fos-GV230), Jund-GV230, cJun-GV230, or empty-GV230 on a Lipofectamine 3000 system (Cat#L3000150, Invitrogen, California, USA) after 12 h. The signals were measured using a dual luciferase reporter assay system (Cat#E1500, Promega, Wisconsin, USA).

### Online Database Analysis

The sequencing results of the *TNF-α* gene were analyzed using the BLAT Tool (UCSC Genome Browser, http:// genome-euro.ucsc.edu/). The putative transcription factors of TNF-α were predicted using the PROMO ALGGEN database (http://alggen.lsi.upc.es/cgibin/promo_v3/promo/promoinit.cgi?dirDB=TF_8.3/) within a dissimilarity margin ≤ 5%.

### Statistical Analysis

GraphPad Prism 5 was used to conduct the statistical analysis. Data are expressed as the mean ± standard deviation (SD) of at least three independent experiments unless otherwise indicated. One-way ANOVA and Tukey’s test were used for comparisons between multiple groups, and two-tailed Student’s *t* test for used for comparisons between two groups. A *P* value of < 0.05 was considered to indicate a statistically significant result.

## Results

### TNF-α Plays an Important Role in the Pathology of Ischemic Stroke

**Fig. 1:**
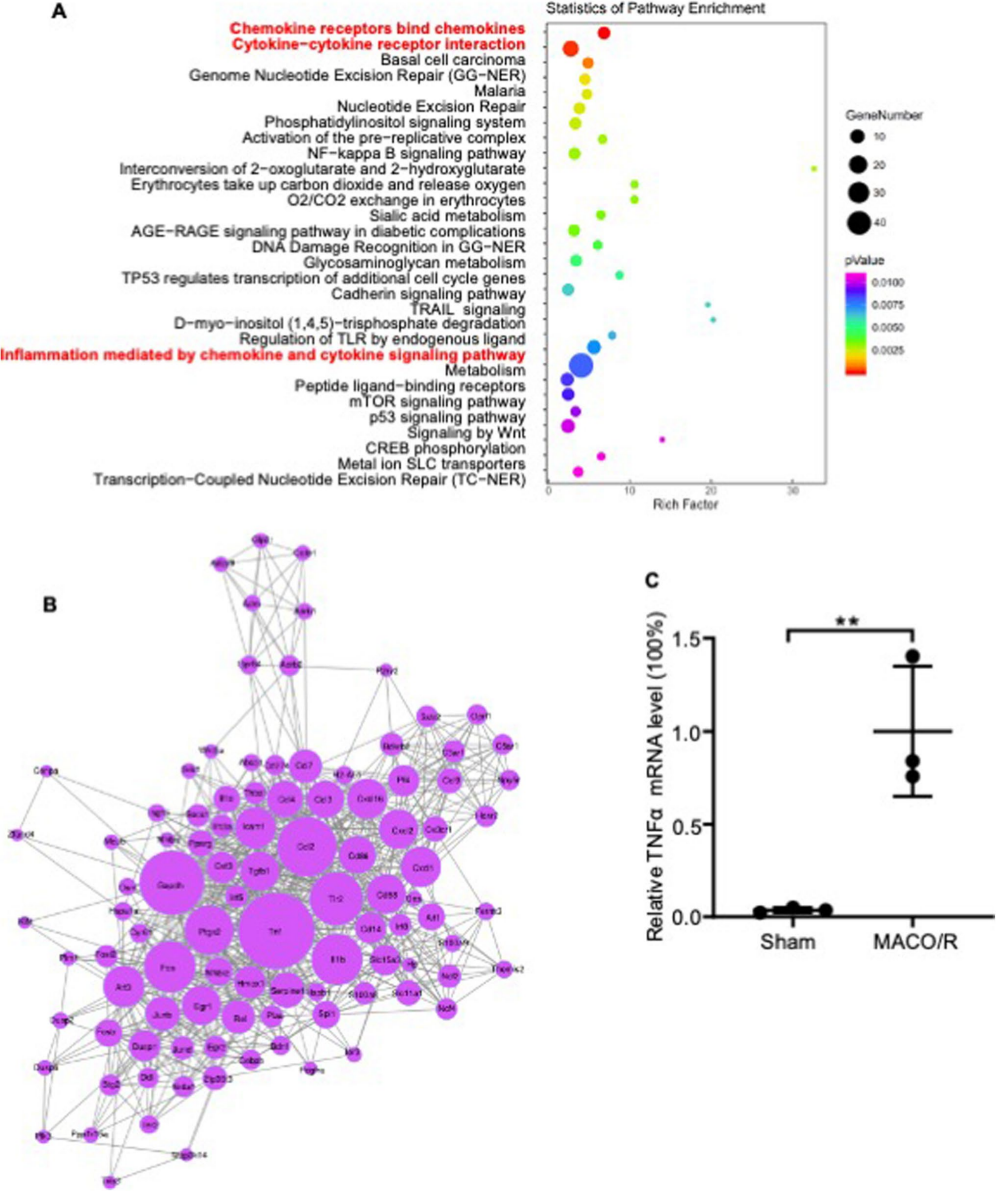
Pathway and protein–protein interaction (PPI) network analysis of genes upregulated in the MCAO/R and control groups. **A** Scatter plot showing enriched pathways of the upregulated genes. The size of each circle indicates the number of significantly upregulated genes in each corresponding pathway. The enrichment factor was calculated using the number of enriched genes divided by the number of all background genes in each pathway. A *P* value of < 0.05 indicates statistical significance. **B** PPI network based on significantly upregulated genes. The size of each circle indicates the node degree of the corresponding gene, and the gray lines represent interacting partners. **C** Quantitative RT-PCR validation of TNF-α mRNA levels between the I/R and control groups. Data are presented as the mean ± SD of two independent experiments. *n* = 9 mice/group. *, *P* < 0.05; **, *P* < 0.01; ***, *P* < 0.001; ****, *P* < 0.0001

**Fig. 2:**
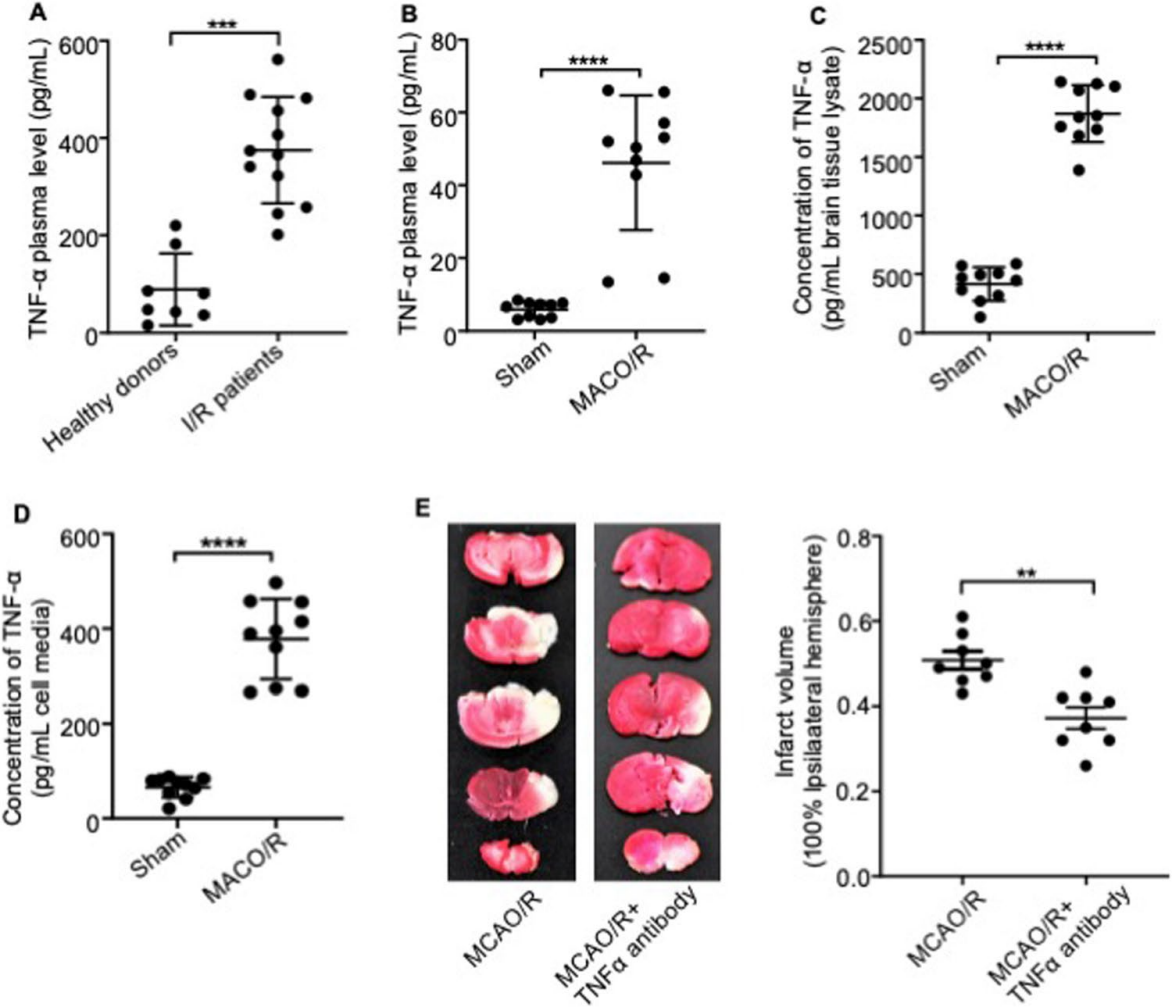
TNF-α plays an important role in the pathology of ischemic stroke. **A** TNF-α plasma levels in heathy donors and stroke patients. **B** TNF-α plasma levels in sham-operated and MCAO model mice. **C** TNF-α concentration in the ischemic cortex of sham-operated and I/R mice. **D** TNF-α levels in the media of primary cultured astrocytes cultured under normal and OGD/R conditions. **E** Left, representative images of TTC-stained brain slices from MCAO/R mice treated with anti-TNF-α antibodies (20 µg/g) or the vehicle at the onset of reperfusion. Right, infarction volume of the groups indicated. Data are presented as the mean ± SD of two independent experiments. *n* = 9 mice/group. *, *P* < 0.05; **, *P* < 0.01; ***, *P* < 0.001; ****, *P* < 0.0001

To determine the transcriptomic profile associated with I/R injury, we sequenced total RNA isolated from the ipsilateral and contralateral cerebral cortices of mice that had undergone MCAO/R. The contralateral undamaged cortices were used as controls. A total of 398 genes showed twofold upregulation, while 231 genes were downregulated in the MCAO/R mice relative to the control (Supplemental Fig. 3A), and upregulated genes chosen at random were validated using qRT-PCR (Supplemental Fig. 3B). Functional annotation of these DEGs indicated that I/R injury activated the inflammatory signaling pathway in the cerebral cortex (Fig. 1A). The protein–protein interaction (PPI) network of the DEGs further indicated the key role played by TNF-α in the pathology of I/R injury (Fig. 1B). Consistent with this finding, TNF-α mRNA expression was significantly upregulated in the brain tissue of mice that had undergone MCAO/R (Fig. 1C). In addition, plasma TNF-α levels were markedly elevated in stroke patients compared with healthy controls (Fig. 2A). We also found that TNF-α of circulating level and brain tissue lysate were significantly increased at 8 h post-reperfusion in the MCAO/R model (Fig. 2B–C). Furthermore, primary astrocytes cultured under OGD/R conditions in vitro to mimic I/R injury also exhibited a significant increase in TNF-α levels (Fig. 2D). To ascertain the pathological role played by TNF-α in acute ischemic stroke, we injected mice with anti-TNF-α antibodies immediately before reperfusion performed 60 min after MCAO. As shown in Fig. 2E, the volume of the infarct region at 12 h post-reperfusion was significantly smaller in mice treated with the anti-TNF-α antibodies compared with the untreated control. Taken together, TNF-α secreted by astrocytes plays an important role in the pathologic progression of ischemic stroke, and TNF-α blockade can attenuate brain edema and infarct volume following transient MCAO.

**Fig. 3:**
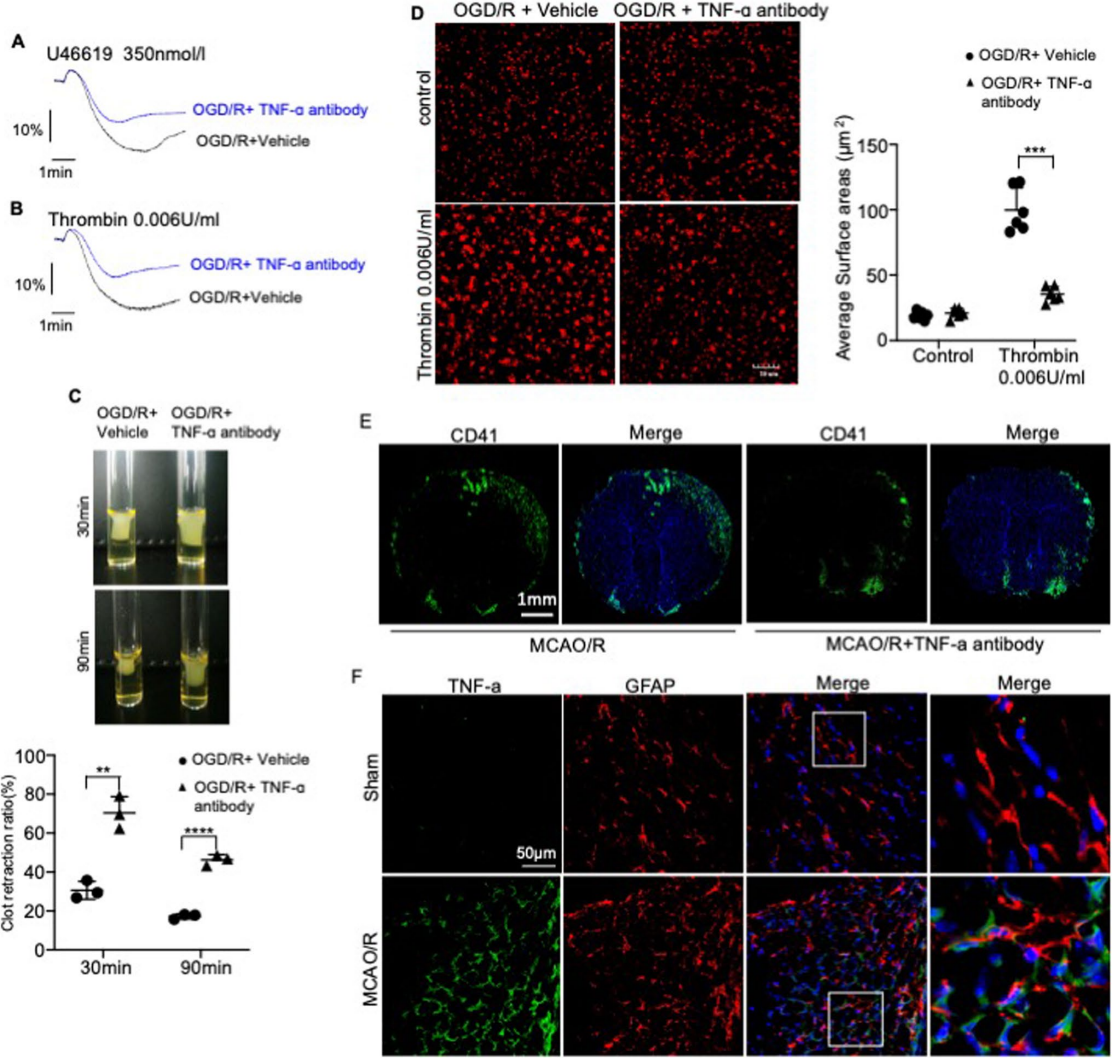
TNF-α released by astrocytes activates platelets. **A**, **B** Aggregation of anti-TNF-α antibody-stimulated platelets in the presence of U46619 (**A**) and thrombin (**B**) at 37 °C. The traces are representative of the results of three independent experiments. Histograms show maximum platelet aggregation under the conditions indicated. **C** Representative images showing the retraction of fibrinogen and thrombin-induced platelet clots pre-incubated with an anti-TNF-α antibody (10 ng/ml) or the vehicle. 2D retraction of clots was measured, and the data are expressed as retraction ratio: 1 − (final clot size/initial clot size). **D** Representative images showing the spread of anti-TNF-α antibody or vehicle-treated platelets on fibrinogen-coated wells in the presence of thrombin or the vehicle at 37 °C for 2 h. Scale bar, 30 μm. Surface areas of single platelets from 10 randomly selected fields of three different tests. **E**, **F** Representative immunofluorescence images showing CD41 + (red), GFAP + (red), and TNF-α + (green) cells in the cerebral cortical tissues of mice subjected to MCAO/R and treated with anti-TNF-α antibody or vehicle at the onset of reperfusion. Scale bar, 1000 μm (**E**); scale bar, 50 μm (**F**). Data are presented as the mean ± SD of two independent experiments. *n* = 9 mice/group. *, *P* < 0.05; **,* P* < 0.01; ***, *P* < 0.001; ****, *P* < 0.0001

### TNF-α Aggravates I/R Injury by Promoting Platelet Aggregation Post-ischemia

We next test whether pretreatment of anti-TNF-α inhibits platelet aggregation induced by thrombin or U46619 in vitro. In vitro, the max platelet aggregation rate (%) in the control group reached 65.43% (U6619-induced) and 70.32% (thrombin-induced) (Fig. 3A–B). All anti-TNF-α antibody-treated groups showed distinct decrease in platelet aggregation versus the control group (*P* < 0.01) in an astrocyte injury model induced by OGD/R (Fig. 3A–B). Integrin αIIbβ3-mediated outside-in signaling can drive clot retraction, and our results demonstrated that the average ratio of clot retraction of PRP was significantly higher in a TNF-α treated astrocyte injury model induced by OGD/R compared with vehicle-treated astrocytes (Fig. 3C). In the presence of thrombin, the area of vehicle-treated platelets undergoing spreading was 80.523 ± 2.641 μm^2^, which was significantly larger than that of TNF-α antibody-treated platelets (26.016 ± 0.513 μm^2^). The TNF-α antibody inhibited platelet spreading in the astrocyte injury model induced by OGD/R compared with vehicle-treated astrocytes (Fig. 3D). Therefore, our results show that TNF-a released by OGD/R-induced astrocytes can activate platelets in vitro.

Given the role played by platelets in the pathogenesis of ischemic stroke, we analyzed the functional relationship between TNF-α secretion and platelet aggregation in vivo. As shown in Fig. 3E, significant levels of platelet accumulation were found in the cerebral cortex of MCAO/R mice compared to that of the sham-operated controls. Furthermore, the cerebral cortex sections stained with antibodies against TNF-α and astrocytes showed that TNF-α released by astrocytes was co-localized with the platelets, indicating that TNF-α can act on platelets to regulate the reactivity of the platelets (Fig. 3F).

**Fig. 4:**
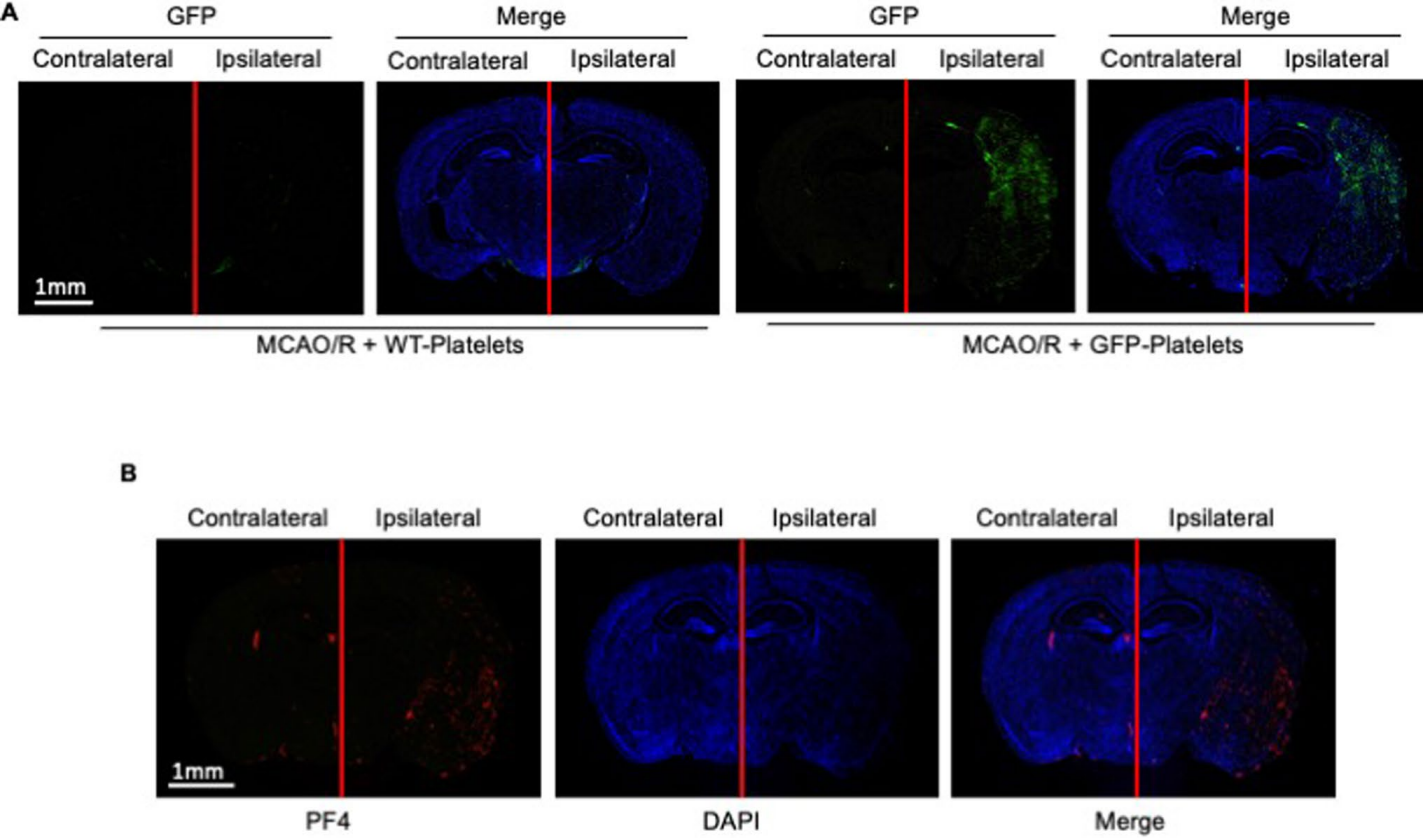
Platelets infiltrating brain tissue during ischemic stroke are activated. Platelets isolated from GFP transgenic or WT mice were pre-incubated for 10 min with TNF-α (10 ng/ml) and then injected to MCAO/R mice at the onset of reperfusion. **A** Representative immunofluorescence images showing GFP + platelets (green) in the cerebral cortical tissues of mice subjected to MCAO/R and treated with TNF-α-activated GFP + platelets or the vehicle at the onset of reperfusion. **B** Representative immunofluorescence images showing PF4 + (red) cells in the cerebral cortical tissues of mice subjected to MCAO/R and treated with TNF-α-activated GFP + platelets or the vehicle at the onset of reperfusion. Scale bar, 25 μm

To confirm whether platelets infiltrating into brain tissue have been activated, we isolated platelets from GFP transgenic mice and injected them into mice with GFP platelets activated ex vivo by TNF-α prior to inducing MCAO/R (Supplemental Fig. 1B). We found GFP-positive platelets in the mouse brain sections after injecting the GFP platelets in MCAO/R using confocal microscopy (Fig. 4A). In addition, we stained the brain sections with an antibody against PF4, a component of platelet α granules released by activated platelets. The expression of PF4 in the ischemic region increased significantly in MCAO/R mice (Fig. 4B). Together, these results indicate that platelets that infiltrate brain tissue have been activated.

### TNF-α Alleviated Platelet Dysfunction Induced by Cerebral I/R Injury Through the RIP1/RIP3/AKT Pathway

**Fig. 5:**
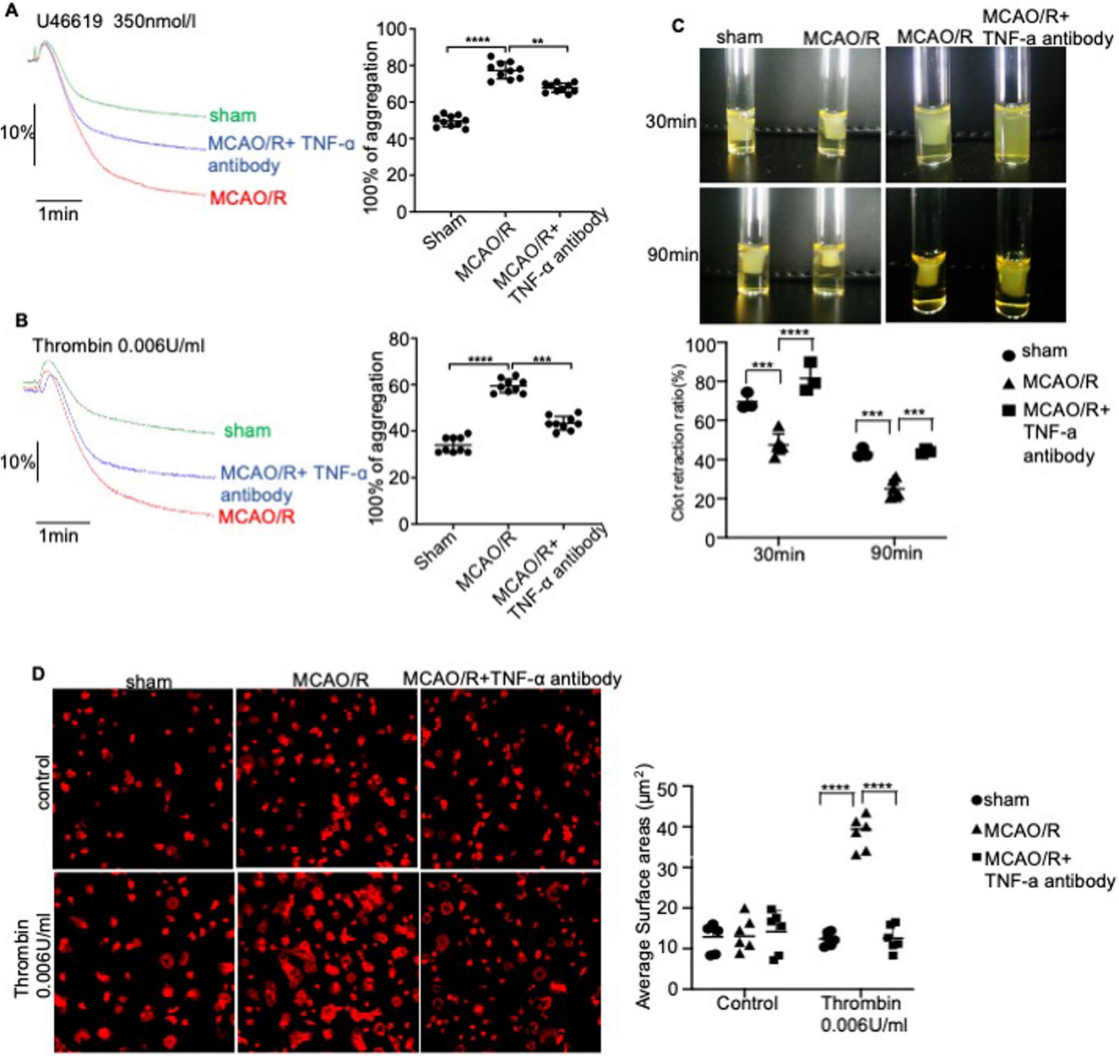
In vivo neutralization of TNF-α blocks platelet aggregation and integrin signaling. **A**, **B** Representative images showing the aggregation of platelets obtained from the sham, I/R, and I/R + anti-TNF-α antibody groups in the presence of **A** U46619 and **B** thrombin at 37 °C. The traces are representative of the results of three independent experiments. The histograms show maximum platelet aggregation under the conditions indicated. **C** Representative images showing the clot retraction of platelets from the sham, I/R, and I/R + anti-TNF-α antibody groups in the presence of fibrinogen and thrombin. 2D retraction of clots was measured, and the data are expressed as retraction ratio: 1 − (final clot size/initial clot size). **D** Representative images showing the spread of platelets from the sham, I/R, and I/R + anti-TNF-α antibody groups on fibrinogen-coated wells in the presence of thrombin or vehicle at 37 °C for 2 h. Surface areas of single platelets from 10 randomly selected fields of three different tests. Scale bar, 30 μm. Data are presented as the mean ± SD of two independent experiments. *n* = 9 mice/group. *, *P* < 0.05; **, *P* < 0.01; ***, *P* < 0.001; ****, *P* < 0.0001

To elucidate the mechanisms by which TNF-α stimulates platelet aggregation in vivo, we induced platelet aggregation using a panel of agonists and found that thrombin- and U46619-induced aggregation increased in PRP of MCAO/R mice. However, TNF-α antibody pretreatment significantly reduced U46619- and thrombin-induced platelet aggregation compared with the vehicle-treated controls, indicating that the blocking of TNF-α may exhibit a specific antiplatelet mechanism of action (Fig. 5A–B). Consistent with ablated platelet thrombin, significantly higher levels of clot retraction were observed in MCAO/R platelets that had recovered by the addition of anti-TNF-α (Fig. 5C). These results suggest that clot retraction requires the inhibition of TNF-α function. We further investigated the role of TNF-α on platelet activation by analyzing platelet spreading. Mouse platelets were incubated on fibrinogen-coated coverslips and stimulated with thrombin. Thrombin significantly stimulated lamellipodia formation in the MCAO/R platelets but not in anti-TNF-α treatment MCAO/R platelets. In the presence of thrombin, the area of MCAO/R platelets undergoing spreading was 38.362 ± 3.589 μm^2^, which was significantly larger than that of anti-TNF-α treatment MCAO/R platelets (10.023 ± 1.953 μm^2^) (Fig. 5D). The results showed that cerebral ischemia/reperfusion injury can promote platelet spreading, and TNF-α neutralization can reverse this process. Thus, these results indicate that cerebral ischemia–reperfusion injury can promote platelet aggregation and the activation of integrin apart from signaling, while TNF-α neutralization can reverse this process.

**Fig. 6:**
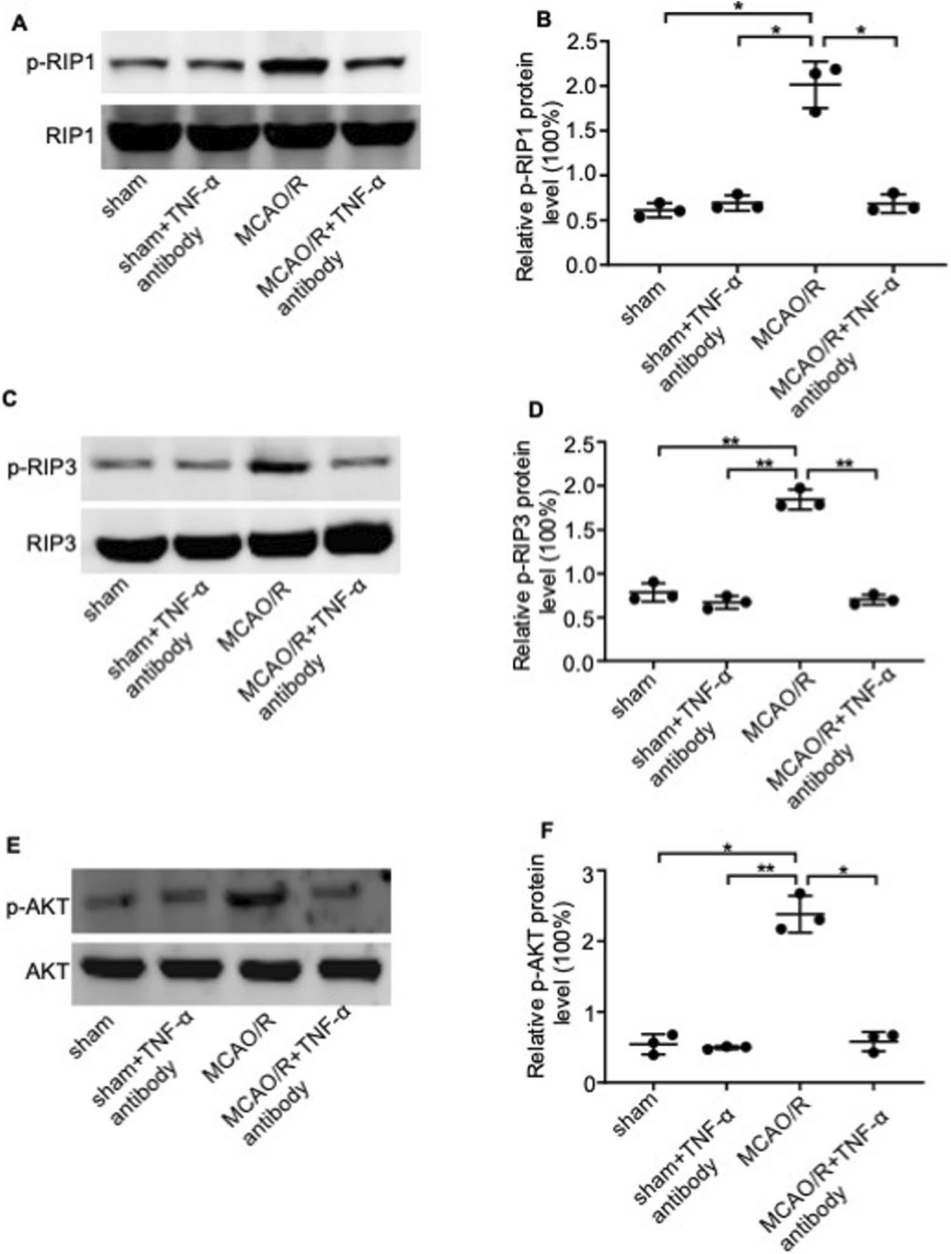
TNF-α regulates the intracellular signaling of platelets. Immunoblots and histograms showing levels of **A**, **B** p-RIP1; **C**, **D** p-RIP3; and **E**, **F** p-AKT in platelets from the sham, I/R, and I/R + anti-TNF-α antibody groups. Data were normalized to the level of the loading control total protein. Data are presented as the mean ± SD of two independent experiments. *n* = 9 mice/group. *, *P* < 0.05; **, *P* < 0.01; ***, *P* < 0.001; ****, *P* < 0.0001

The PI3K-AKT signaling pathway plays an essential role in regulating the second wave of ADP secretion in platelets in response to U46619. We found that TNF-α upregulated levels of p-RIP1 and p-RIP3 and induced AKT phosphorylation (Fig. 6A–C). Taken together, our results indicate that TNF-α released by astrocytes following I/R injury leads to platelet invasion and aggregation in the ischemic area of the cerebral cortex by activating the RIP1/RIP3/AKT pathway.

### TNF-α-Activated Platelets Aggravate Ischemia/Reperfusion Injury in Mice

**Fig. 7:**
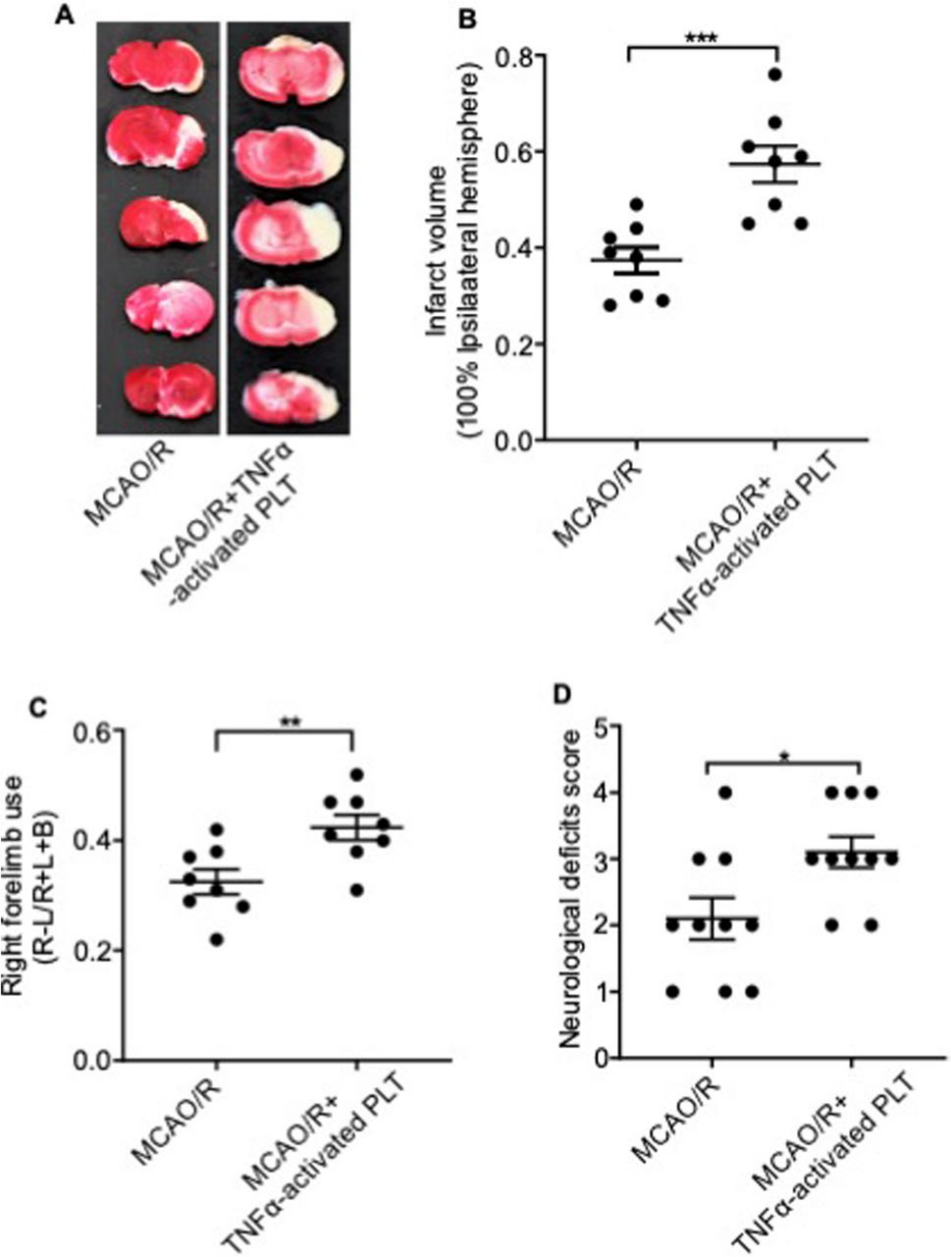
In vivo injection of TNF-α-activated platelets aggravates the infarction volume and neurologic deficits. **A** Representative images of TTC-stained cortical tissues, **B** infarct volume, **C** cylinder test results, and **D** neurological scores of mice subjected to MCAO/R and treated with anti-TNF-α antibody or vehicle at the onset of reperfusion. Data are presented as the mean ± SD of two independent experiments.* n* = 9 mice/group. *, *P* < 0.05; **, *P* < 0.01; ***, *P* < 0.001; ****, *P* < 0.0001

To determine whether TNF-α-induced platelet aggregation in the cerebral cortex was the direct cause of ischemic injury, we injected mice with platelets activated ex vivo with TNF-α (data not shown) before MCAO/R induction. As shown in Fig. 7A–B, the volume of the ischemic region was significantly greater in the activated platelet-treated mice compared with untreated MCAO mice. In addition, the use of the right forelimb and neurological deficit scores were also significantly higher in mice treated with TNF-α-activated platelets compared with the vehicle-treated group (Fig. 7C–D).

Thus, the invasion and accumulation of TNF-α-activated platelets into the ischemic area of the cortex mediate the pathological progression of stroke.

### AP-1 Regulated the Expression of TNF-α in I/R-Injured Mice Cortex

**Fig. 8:**
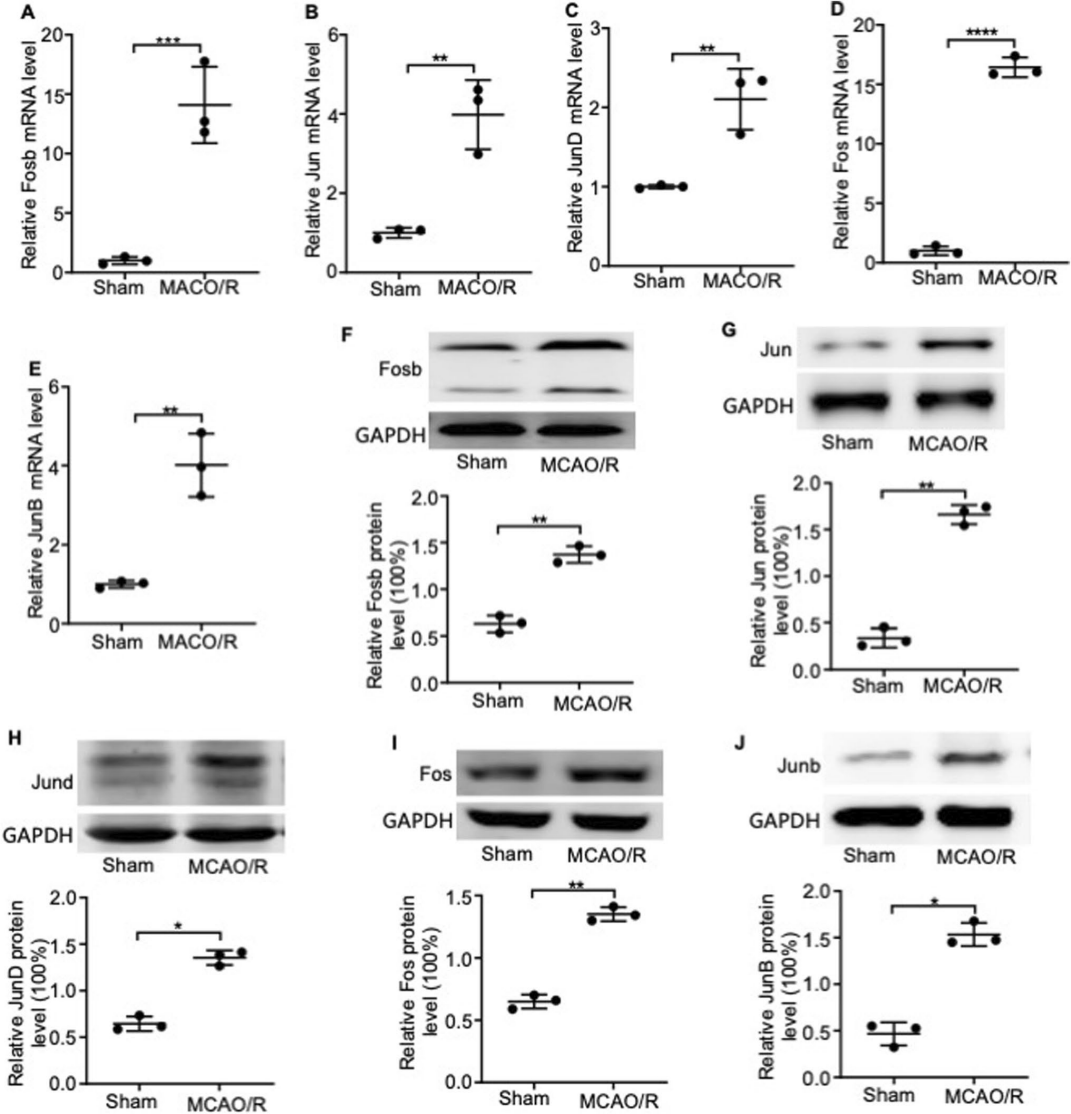
Effect of I/R on AP-1 gene and protein expression in the cerebral cortex. **A** FosB, **B** Jun, **C** JunD, **D** Fos, and **E** JunB mRNA levels in the ipsilateral and contralateral cortices of the control and MCAO/R groups. Data is normalized to GAPDH levels. Fold changes are shown. **F** FosB, **G** Jun, **H** JunD, **I** Fos, and **J** JunB protein levels in the groups indicated. Data are presented as the mean ± SD of two independent experiments. *n* = 9 mice/group. *, *P* < 0.05; **, *P* < 0.01; ***, *P* < 0.001; ****, *P* < 0.0001

**Fig. 9:**
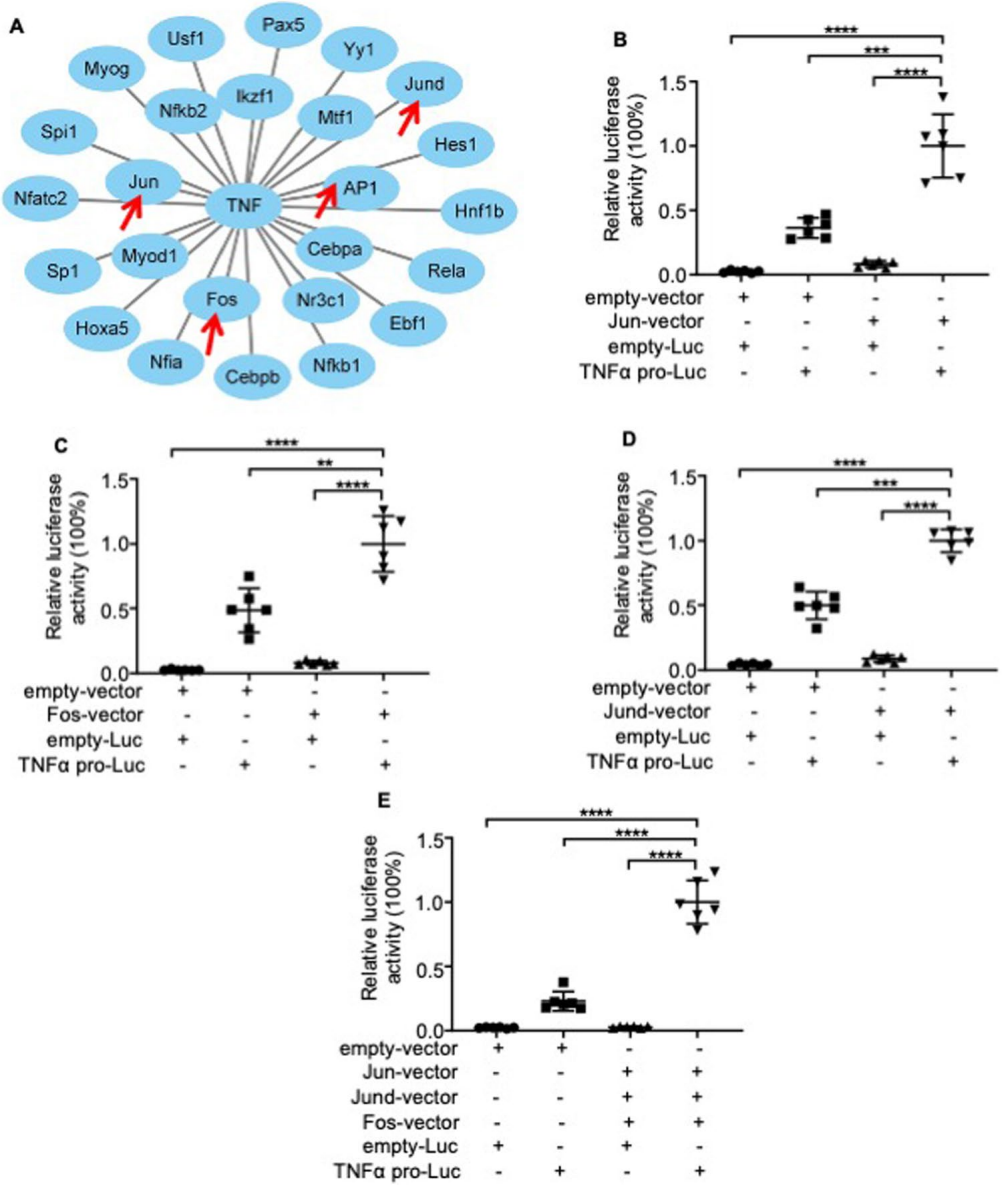
AP-1 regulates the expression of TNF-α. **A** Transcription factors that target TNF-α were predicted using the PROMO ALGGEN database within a dissimilarity margin less than or equal to 5%. **B**–**D** Luciferase activity levels of HEK293T cells co-transfected with the TNF-α promoter-Luc reporter and **B** GV230-Jun, **C** GV230-Fos, or **D** GV230-JunD or empty vector. **E** Luciferase activity levels of HEK293T cells co-transfected with the TNF-α promoter-Luc reporter and GV230-vector containing Jun, Fos, and JunD or an empty vector as the control. Data are presented as the mean ± SD of two independent experiments. *n* = 9 mice/group. *, *P* < 0.05; **,* P* < 0.01; ***, *P* < 0.001; ****, *P* < 0.0001

To gain further insights into the regulation of TNF-α in the I/R-injured cortex, we screened RNA-seq data for upregulated transcription factors in the MCAO/R model, and detected the significant upregulation of the members of the activator protein (AP)-1 family, which includes cFos, FosB, cJun, JunB, and JunD (Supplemental Fig. 4 and Supplementary Table 2). Further analysis of the cortical tissue indicated a marked increase at both mRNA and protein level following I/R injury (Fig. 8). We also predicted the TNF-α transcription factors using the UCSC website (http://genome.ucsc.edu/) and an online bioinformatics tool, the PROMO ALGGEN database (http://alggen.lsi.upc.es/cgibin/promo_v3/promo/promoinit.cgi?dirDB=TF_8.3/) and found significant overlapping between putative transcription factors and those upregulated in the I/R-injured cortex (Fig. 9A). Therefore, we hypothesized that AP-1 is the critical transcriptional regulator of TNF-α during I/R injury. To determine whether TNF-α is indeed the target gene of AP-1, we performed a luciferase report assay to detect the binding of Jun, Jund, and Fos to the TNF-α promoter. As shown in Fig. 9B–E, the luciferase activity of the reporter gene placed under the TNF-α promoter increased following co-transfection with Jun, Jund, and/or Fos expressing vectors. Taken together, our results indicate that AP-1 transcriptionally upregulates TNF-α in the mouse cortex following I/R injury by directly binding to its promoter region.

## Discussion

**Fig. 10:**
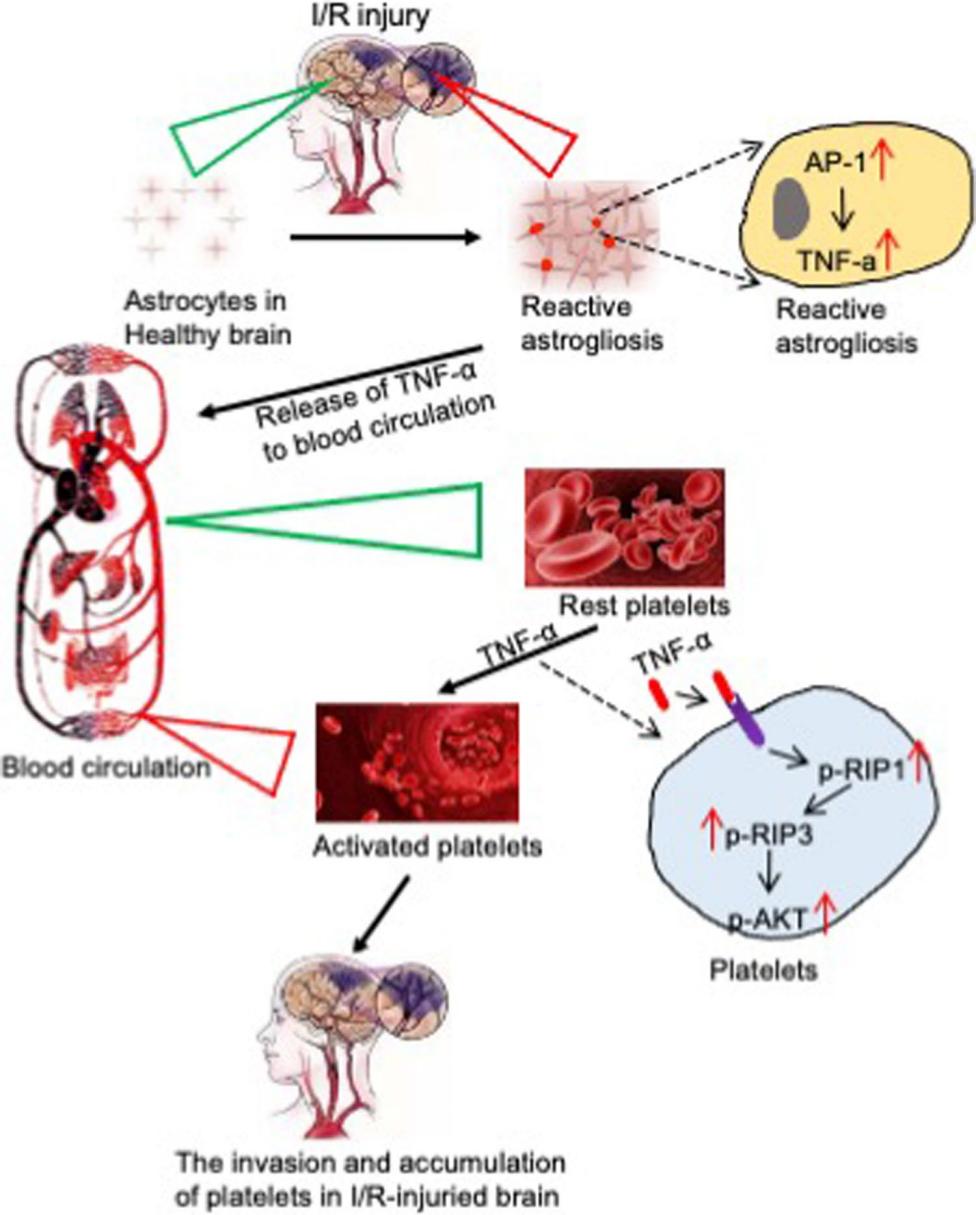
Schematic diagram of the hypothesis indicating that platelets participate in the pathology of ischemic stroke. Ischemic stroke leads to astrogliosis in the cerebral cortex, while AP-1 transcriptionally upregulates TNF-α, and TNF-α released by activated astrocytes induces high platelet reactivity through the RIP1/RIP3/AKT pathway. These platelets eventually invade and accumulate in the ischemic area, which aggravates the pathological progression of ischemic stroke

Our findings indicate that ischemic stroke leads to astrogliosis in the cerebral cortex, while AP-1 transcriptionally upregulates TNF-α, and that TNF-α released by activated astrocytes induces high platelet reactivity through the RIP1/RIP3/AKT pathway. These platelets eventually invade and accumulate in the ischemic area, which aggravates the pathological progression of ischemic stroke (Fig. 10).

### TNF-α from Astrocytes Plays an Important Role in the Pathology of Ischemic Stroke

Inflammation is an independent risk factor for ischemic stroke [20], and high levels of inflammatory cytokines in the ischemic region increase the risk of stroke [21]. Furthermore, previous studies have found associations between the genetic polymorphisms of TNF-α and cerebrovascular disease susceptibility. For instance, Um et al. reported on the etio-pathological role of TNF-α-308G/A in cerebral infarction [22], while another study showed that TNF-α gene variants are a risk factor for ischemic stroke in the Korean population [23]. Consistent with the results of these studies, we conducted transcriptome sequencing of the murine cerebral cortex with I/R injury and found that genes involved in the inflammatory signaling pathway were upregulated. Furthermore, TNF-α levels in the cerebral cortex and serum were also significantly higher after MCAO/R, and the administration of an anti-TNF-α antibody protected mice from I/R injury. These findings clearly establish the pivotal role played by TNF-α in the progression of ischemic stroke.

Astrocytes are the most abundant cells in the cerebral cortex and the major inflammatory fraction. Neuroinflammation induced by activated astrocytes contributes substantially to ischemic stroke injury. Ischemic stroke injury can activate astrocytes, causing them to produce and release excessive amounts of TNF-α. We found that anti-TNF-α treatment significantly inhibited the hyperactivation of astrocytes and significantly decreased the pathologic progression of MCAO/R injury. However, the underlying mechanism remains unclear.

### TNF-α Aggravates I/R Injury by Promoting Platelet Aggregation Post-ischemia Through the RIP1/RIP3/AKT Pathway

Platelets are crucially involved in inflammation as well as a key factor in the development and progression of ischemic stroke. Inappropriate activation of platelets leads to thrombosis and vessel occlusion which will finally result in stroke. Antiplatelet therapies are an important strategy in stroke management. When different platelet inhibition mechanisms are used, combined antiplatelet therapy may have important benefits in the prevention of ischemic stroke injury. Pro-coagulant factors, such as thrombin, GPVI, and ROS, play critical roles in mediating cerebral I/R injury [7, 24, 25]. We showed that TNF-α released by astrocytes induced platelet invasion into the cerebral cortex of MCAO/R mice, which was alleviated by pre-treatment with a TNF-α antibody. We found that anti-TNF-α treatment inhibited platelet clotting, platelet aggregation in response to thrombin, and platelet spreading on a collagen surface.

TNF-α is known to activate RIP3 [12], while RIP1 can dissociate from complex I and bind to RIP3 to form a new protein complex necrosome in response to TNF stimulation[26]. RIP3 lies upstream of AKT and regulates platelet activation [27]. Insoluble RIP1, RIP3, and PI3K levels increased in the infarct area of mice suffering from MCAO/R injury [28]. Previous studies have reported that necrostatin-1 protects against cerebral injury after acute ischemic stroke by preventing RIP1/RIP3/PI3K-mediated necroptosis[29]. Consistent with these studies, we found that TNF-a binds to its receptor, TNFR, to trigger downstream signal transmission and activate RIP3 and RIP1 in ischemic stroke. As an important protein kinase, PI3K is known to activate its substrate protein, AKT, which then mediates cell survival and proliferation [30]. In our study, RIP3 activated PI3K by activating the AKT–AKT signaling pathway, which in turn promoted platelet activation. The results of our study indicate that TNF-α released by astrocytes promote platelet aggregation in the cerebral cortex during ischemic stroke by activating the TNFR/RIP1/RIP3/AKT pathway.

Taken together, these findings suggested that TNF-α neutralization has clinical therapeutic significance, so it is necessary to develop TNF-α antibody which can treat the prognosis of cerebral ischemia.

### AP-1 Regulated the Expression of TNF-α in I/R-Injured Mice Cortex

The transcription factor, AP-1, regulates the expression of multiple inflammatory genes, including TNF-α [31]. Mutations in the AP-1 binding site in the TNF-α promoter markedly downregulate the expression of the TNF-α gene [32]. Consistent with these reports, we found that AP-1 can bind to the TNF-α promoter to enhance its expression in the I/R-injured mice cortex. Therefore, we hypothesized that AP-1 may be a critical transcriptional regulator of TNF-α in the MCAO/R-induced cortex.

In summary, TNF-α promotes platelet invasion and aggregation in the ischemic cortex, which aggravates symptoms of stroke. In addition, the neutralization of TNF-α is an effective therapeutic strategy against ischemic stroke that may be used as a potentially novel mechanism to provide protection for platelets during ischemic stroke.

## Supplementary Information

Below is the link to the electronic supplementary material.

Supplementary file1 (DOC 17 KB)

Supplementary file2 Ischemic stroke induction using MCAO andreperfusion (JPEG 37 KB)

Supplementary file3 *In vitro *neutralizationof TNF-αblocks platelet aggregation and integrin signaling (JPEG 21 KB)

Supplementary file4 Differentially expressed genes(DEGs) between the ipsilateral and contralateral cerebral cortexes in I/R mice. (A) Principal components analysis of the samplesfor RNA-seq. Principal Component 1 (PC1, x-axis) represents 36.94%, and PC2(y-axis) represents 19.58% of the total variation in the data. (B) Heatmapshowing upregulated (red) and downregulated (green) genes. (C) Validation ofRNA-Seq data of selected DEGs by qRT-PCR. Thedata represent mean ± SD oftwo independent experiments, n = 9 mice/group. *, P < 0.05; **, P <0.01; ***, P < 0.001; ****P < 0.0001. (JPEG 53 KB)

Supplementary file4 Heatmap showing the up-regulated transcription factors between theipsilateral and contralateral cortices in I/Rmice. (JPEG 48 KB)

Supplementary file6 Primer sequences (JPEG 210 KB)

Supplementary file7 Transcriptome profile ofcerebral cortices from MCAO/R model (JPEG 235 KB)

## Data Availability

The datasets generated and/or analyzed during the current study are available upon reasonable request.
