# Supplementary material for: Astrocyte-Derived TNF-α-Activated Platelets Promote Cerebral Ischemia/Reperfusion Injury by Regulating the RIP1/RIP3/AKT Signaling Pathway
Source: Mol Neurobiol. 2022 Jul 4;59(9):5734–49. doi: 10.1007/s12035-022-02942-z (PMC9395439; doi:10.1007/s12035-022-02942-z)
Supplement: Supplementary file 1 — Supplementary file1 (DOC 17 KB) [file 12035_2022_2942_MOESM1_ESM.docx]

Supplemental Information

**Astrocyte-derived TNF-α Activated platelets promote cerebral**

**ischemia/reperfusion injury by regulating RIP1/RIP3/AKT signaling pathway**

Wei Li^1^, Dengping Liu^2^, Jiaqi Xu^3^, Jun Zha^4^, Chen Wang^4^, Jianzhong An^2^, Zhanli Xie^2^, Shigang Qiao^2,4^

^1^ Cyrus Tang Hematology Center, Soochow University, Suzhou, China

^2^Institute of Clinical Medicine Research, Suzhou Science & Technology Town Hospital, Gusu School, Nanjing Medical University, Suzhou, China

^3^ Nursing Department, Suzhou Science & Technology Town Hospital, Gusu School, Nanjing Medical University, Suzhou, China

^4^ Faculty of Anesthesiology, Suzhou Science & Technology Town Hospital, Gusu School, Nanjing Medical University, Suzhou, China

Correspondence to: Zhanli Xie, Tel: (+86) 19951296975. E-mail [zhanlixie1989@njmu.edu.cn](http://mail.njmu.edu.cn/coremail/XT3/oab/userdetail.jsp?sid=BAHzqjBBwPKOitUEZuBBWFqWmXOndMag&urlfrom=..%2foab%2flist.jsp%3fsid%3dBAHzqjBBwPKOitUEZuBBWFqWmXOndMag%26dn%3da%252f560&dn=a%2f560&uid=zhanlixie1989@njmu.edu.cn); Or Shigang Qiao, Tel: (+86)17715187396. E-mail qiaos@njmu.edu.cn; Institute of Clinical Medicine Research, Suzhou Science & Technology Town Hospital, Gusu School, Nanjing Medical University, 1 Li Jiang Road, Suzhou, 215153, China.

**Figure legends**

**Supplemental Figure 1. Ischemic stroke induction using MCAO and reperfusion**

**Supplemental Figure 2. *In vitro* neutralization of TNF-α blocks platelet aggregation and integrin signaling.**

**Supplemental Figure 3. Differentially expressed genes (DEGs) between the ipsilateral and contralateral cerebral cortexes in I/R mice.** (A) Principal components analysis of the samples for RNA-seq. Principal Component 1 (PC1, x-axis) represents 36.94%, and PC2 (y-axis) represents 19.58% of the total variation in the data. (B) Heatmap showing upregulated (red) and downregulated (green) genes. (C) Validation of RNA-Seq data of selected DEGs by qRT-PCR. The data represent mean ± SD of two independent experiments, n = 9 mice/group. *, P < 0.05; **, P < 0.01; ***, P < 0.001; ****P < 0.0001.

**Supplemental Figure 4. Heatmap showing the up-regulated transcription factors between the ipsilateral and contralateral cortices in I/R mice.**

**Supplemental Table 1. Primer sequences**

**Supplemental Table 2. Transcriptome profile of cerebral cortices from MCAO/R model**
