# Supplementary figures and images for: Astrocyte-Derived TNF-α-Activated Platelets Promote Cerebral Ischemia/Reperfusion Injury by Regulating the RIP1/RIP3/AKT Signaling Pathway
Source: Mol Neurobiol. 2022 Jul 4;59(9):5734–49. doi: 10.1007/s12035-022-02942-z (PMC9395439; doi:10.1007/s12035-022-02942-z)

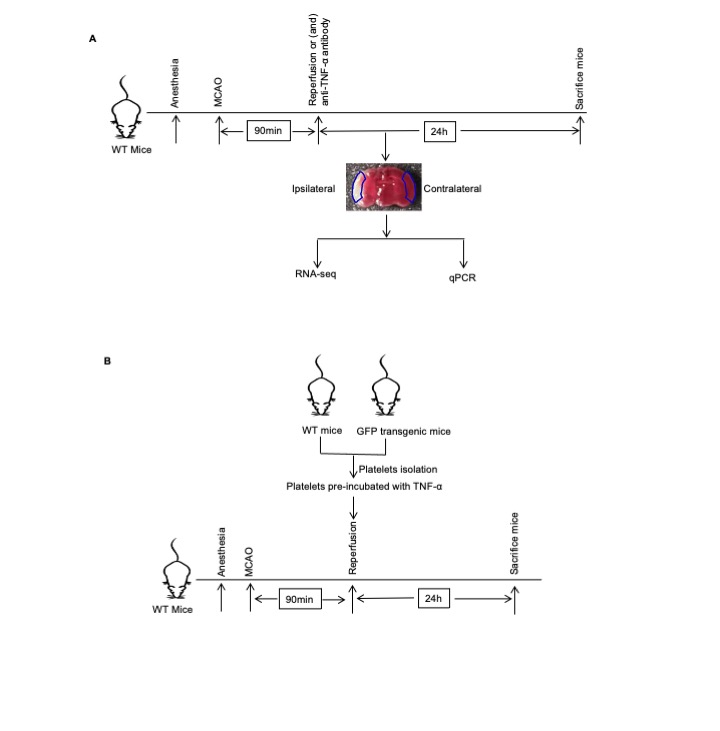

Supplement: Supplementary file 2 — Supplementary file2 Ischemic stroke induction using MCAO andreperfusion (JPEG 37 KB) [file 12035_2022_2942_MOESM2_ESM.jpeg]

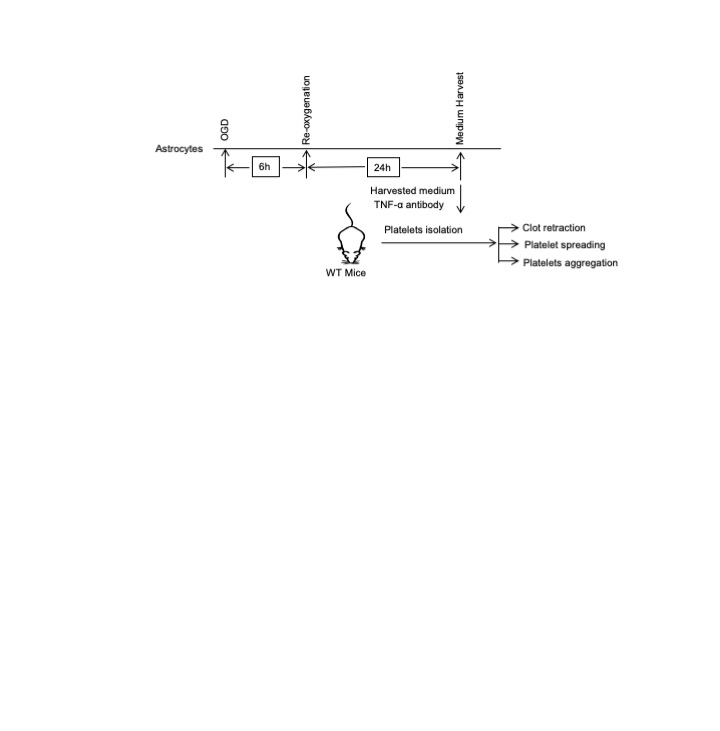

Supplement: Supplementary file 3 — Supplementary file3 In vitro neutralizationof TNF-αblocks platelet aggregation and integrin signaling (JPEG 21 KB) [file 12035_2022_2942_MOESM3_ESM.jpeg]

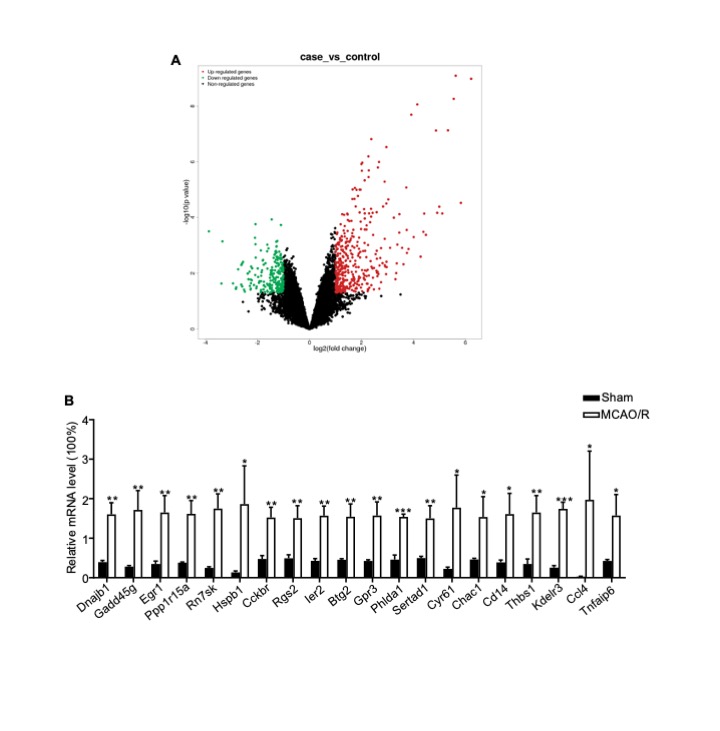

Supplement: Supplementary file 4 — Supplementary file4 Differentially expressed genes(DEGs) between the ipsilateral and contralateral cerebral cortexes in I/R mice. (A) Principal components analysis of the samplesfor RNA-seq. Principal Component 1 (PC1, x-axis) represents 36.94%, and PC2(y-axis) represents 19.58% of the total variation in the data. (B) Heatmapshowing upregulated (red) and downregulated (green) genes. (C) Validation ofRNA-Seq data of selected DEGs by qRT-PCR. Thedata represent mean ± SD oftwo independent experiments, n = 9 mice/group. *, P < 0.05; **, P <0.01; ***, P < 0.001; ****P < 0.0001. (JPEG 53 KB) [file 12035_2022_2942_MOESM4_ESM.jpeg]

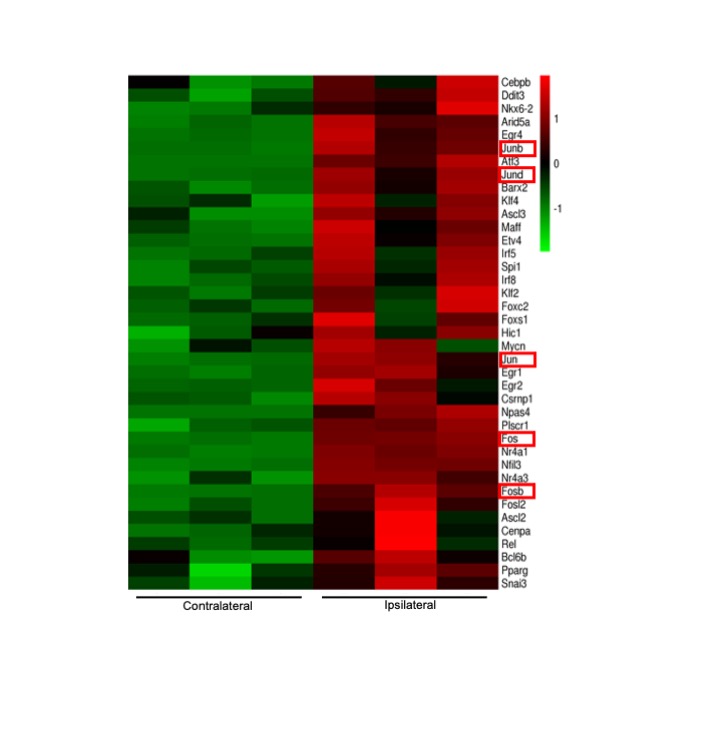

Supplement: Supplementary file 5 — Supplementary file4 Heatmap showing the up-regulated transcription factors between theipsilateral and contralateral cortices in I/Rmice. (JPEG 48 KB) [file 12035_2022_2942_MOESM5_ESM.jpeg]

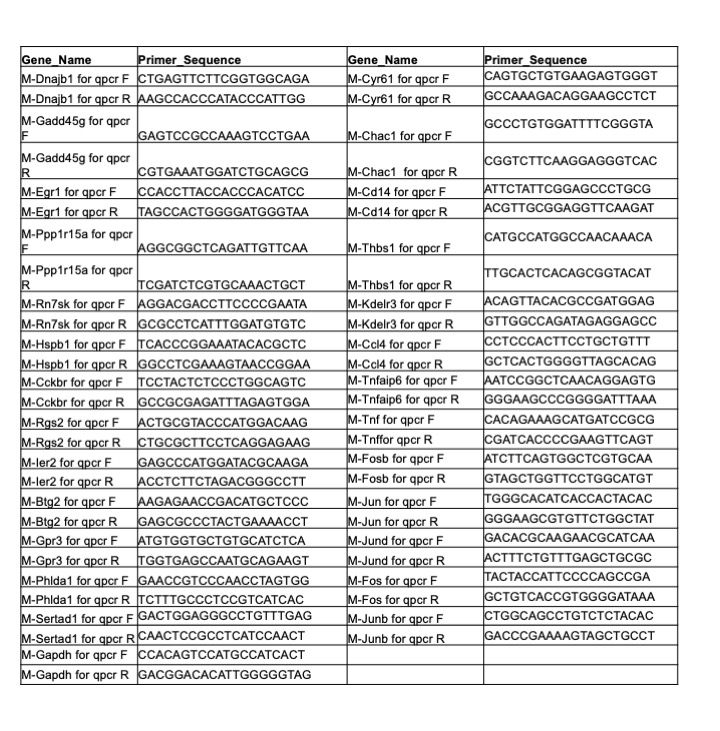

Supplement: Supplementary file 6 — Supplementary file6 Primer sequences (JPEG 210 KB) [file 12035_2022_2942_MOESM6_ESM.jpeg]

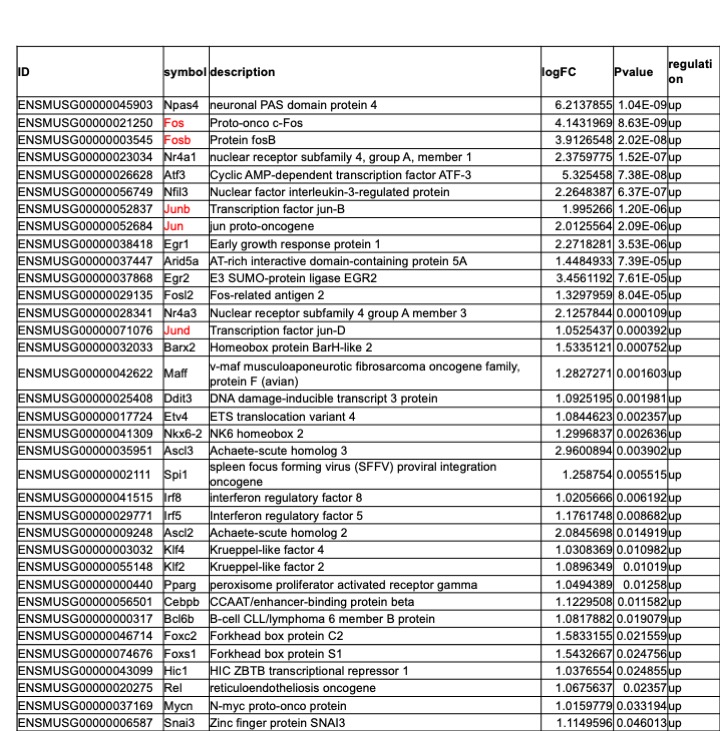

Supplement: Supplementary file 7 — Supplementary file7 Transcriptome profile ofcerebral cortices from MCAO/R model (JPEG 235 KB) [file 12035_2022_2942_MOESM7_ESM.jpeg]
